# Changes in Immune‐Inflammation Status and Acute Ischemic Stroke Prognosis in Prospective Cohort

**DOI:** 10.1002/acn3.70252

**Published:** 2025-11-24

**Authors:** Songfang Chen, Wenting Huang, Yitian Liu, Xuan Chen, Buyu Ke, Qian Shen, Hanyu Cai, Jing Sun, Yan Li, Yungang Cao, Beilei Hu, Keyang Chen

**Affiliations:** ^1^ Department of Neurology, The Second Affiliated Hospital and Yuying Children's Hospital of Wenzhou Medical University, Wenzhou China; ^2^ Wenzhou Key Laboratory of Neurogenetics Wenzhou China; ^3^ Department of Neurology The First Affiliated Hospital of Wenzhou Medical University Wenzhou China; ^4^ Wenzhou Medical University Wenzhou China

**Keywords:** association, dynamic change, immune‐inflammation, ischemic stroke, prognosis

## Abstract

**Background:**

Inflammation is a critical risk factor for poor outcomes in cerebral infarction. Prior studies focused primarily on baseline inflammation status, neglecting dynamic longitudinal changes. We try to investigate the association between immune‐inflammation status alterations and stroke prognosis, and evaluated three systemic biomarkers' predictive efficacy.

**Methods:**

In this prospective cohort study of ischemic stroke patients, C‐reactive protein‐albumin‐lymphocyte (CALLY), Systemic Immune‐Inflammation Index (SII), and Systemic Inflammation Response Index (SIRI) were assessed. Immune‐inflammation changes were measured between baseline and a second survey. The primary outcome was death or major disability (modified Rankin Scale score ≥ 3) after stroke onset. Prognostic performance for poor outcomes was evaluated at baseline. The association was calculated by Cox proportional hazard models.

**Results:**

Elevated baseline CALLY correlated with reduced poor outcome risk (OR 0.33, 95% CI 0.20–0.53), while higher SII and SIRI indicated increased risk (SII OR 2.54, 95% CI 1.61–4.06; SIRI OR 2.37, 95% CI 1.47–3.87). Adding these markers to conventional risk factors significantly improved prediction, with CALLY showing the greatest enhancement (*C*‐statistic 0.882 vs. 0.862; *p* = 0.004; NRI = 29.68%; IDI = 0.39%). Compared to stable mild status, progression to severe status increased poor outcome risk, while recovery to mild status reduced risk (CALLY OR 14.2, 95% CI 2.31–64.27; SII OR 1.27, 95% CI 1.01–7.10; SIRI OR 4.10, 95% CI 1.61–10.71). High inflammatory indices and significant changes predicted poor outcomes.

**Conclusions:**

CALLY demonstrated superior predictive ability for ischemic stroke outcomes versus SII/SIRI. Both baseline levels and dynamic changes in immune‐inflammation status significantly correlated with prognosis, suggesting these biomarkers could valuably predict outcomes and guide intervention.

## 1 Introduction

Millions of people worldwide suffer from stroke each year, making it a leading cause of long‐term disability and death [1, 2]. Ischemic stroke accounts for 70% of all stroke cases, and presents a significant clinical challenge [3]. A deeper understanding of the immune‐inflammation response after ischemic stroke is essential for investigating adverse outcomes.

Systemic immune‐inflammation is strongly linked to atherosclerosis, cardiovascular disease (CVD), and an increased risk of all‐cause mortality [4]. Traditional inflammatory markers such as white blood cell count and its subtypes have been shown to predict the risk of CVD [5, 6]. Furthermore, inflammation‐related indices incorporating immune information, such as the Systemic Immune‐Inflammation Index (SII) and Systemic Inflammation Response Index (SIRI), have been shown to predict CVD prognosis and mortality more effectively than individual markers [7, 8]. These indices reflect the balance between inflammation and the immune response, offering a more comprehensive understanding of the immune‐inflammation state. Specifically, SII has been shown to predict poor outcomes in patients with acute myocardial infarction [9], hypertension [10] and acute ischemic stroke with intravenous thrombolysis [11]. The SIRI has been identified as an independent predictor of major poor cardiovascular events in patients undergoing percutaneous coronary intervention [12]. Additionally, the C‐reactive protein‐albumin‐lymphocyte (CALLY) score, which integrates immune, nutritional, and inflammatory status, reflects the overall immune‐nutritional condition, with higher scores indicating better status, lower systemic inflammation, and an improved prognosis in cancer [13]. However, its relevance to cardiovascular and cerebrovascular outcomes remains unclear.

While these studies primarily focused on the baseline inflammatory status, they did not consider changes in the immune‐inflammation state over time. Compared to a single assessment of the body's condition at baseline, studying changes in physical condition can reflect a more comprehensive biological association with disease [14, 15]. Investigating such changes could offer a more detailed understanding of the relationship between the inflammatory progression and stroke prognosis. Therefore, it is urgent to investigate the associations of changes in immune‐inflammation status with stroke outcomes.

In the current study, we utilize three indices to assess the relationship between baseline immune‐inflammation status and stroke prognosis, and evaluate the predictive capacity of these indices. Furthermore, we investigate the association between changes in immune‐inflammation status and both short‐term and long‐term stroke outcomes.

## 2 Methods

### 2.1 Study Design and Population

Participants in this study were drawn from the Stroke and Fibroblast Growth Factor Biomarker Cohort Study (SFBCS), a multicenter prospective study [16]. Adhering to the Strengthening the Reporting of Observational Studies in Epidemiology (STROBE) guidelines [17], the study included 1068 stroke patients or transient ischemic attack (TIA) aged 18 years or older admitted between March 1, 2021, and August 31, 2022. Acute ischemic stroke was diagnosed according to the World Health Organization criteria and confirmed using brain MRI. TIA was defined as a transient episode of neurological dysfunction without evidence of acute infarction on neuroimaging [18]. Follow‐up assessments were conducted at 3 months and 1 year. Patients were excluded if they met any of the following criteria: missing measurements of height, weight, laboratory parameters required to calculate immune‐inflammation scores; with a medical history of cancer; or infection within 2 weeks before admission. Data from the first day and the seventh day of hospitalization were used to assess the changes in immune‐inflammation status. The study was approved by the Ethics Committee of Wenzhou Medical University and institutional review boards and all participating hospitals, and written informed consent was obtained from all participants. The trial is registered at http://www.chictr.org.cn (ChiCTR2100051104).

### 2.2 Assessment of Immune‐Inflammation Status

We used SII, SIRI, and CALLY indices to assess the immune‐inflammation state. SII is calculated as the product of peripheral blood platelet and neutrophil counts divided by lymphocyte count, whereas SIRI is the product of monocyte and neutrophil counts divided by lymphocyte count. The CALLY score integrates immune, nutritional, and inflammatory markers to provide a comprehensive evaluation of immune‐inflammation status [19], which is calculated using the formula (albumin × lymphocyte)/(CRP × 10^4^), where the product of lymphocyte count and albumin is divided by CRP and multiplied by 10,000. Each immune‐inflammation index was categorized into three levels (mild, moderate, and severe) based on the tertiles of the distribution in the overall study population: the lowest third was classified as mild, the middle third as moderate, and the highest third as severe. The second index values were classified into the same categories using the cut‐offs from the first index, and the relationship between longitudinal changes in the indices and prognosis was assessed.

### 2.3 Data Acquisition

Baseline patient data were collected from electronic medical records, including demographic information (age, sex, body mass index), smoking and alcohol use status, medical history (hypertension, diabetes, hyperlipidemia, previous stroke, coronary heart disease), and baseline stroke severity (modified Rankin Scale [mRS] score and National Institutes of Health Stroke Scale [NIHSS] score). Smoking was defined as consuming at least one cigarette per day, and alcohol consumption was defined as drinking at least 80 g of liquor daily based on a standardized questionnaire. Laboratory data were collected from participating hospitals, including neutrophils, lymphocytes, platelets, monocytes, albumin, and C‐reactive protein (CRP). All laboratories followed standardized testing methods with centralized quality control performed. Dyslipidemia was defined according to the Chinese guidelines for the prevention and treatment of dyslipidemia [20].

### 2.4 Outcome Assessment

Participants were followed up at 3 months and 1 year after stroke onset by trained neurologists. The primary outcome was a composite poor event, including death and severe disability (modified Rankin Scale [mRS] score, 3–6). Secondary outcomes included death (mRS score 6) and major disability. Mortality and vascular event information were obtained from the hospital records or government departments.

### 2.5 Statistical Analyses

Descriptive statistics were used to summarize the data; continuous variables were expressed as mean (standard deviation [SD]) or median (interquartile range [IQR]), while categorical variables were presented as counts (percentages). Multivariable logistic regression and Cox proportional hazards regression models were employed to calculate odds ratios (ORs) with 95% confidence intervals (CIs) for poor outcomes, as well as hazard ratios (HRs) with 95% CIs for mortality and composite vascular events. The proportional hazards assumption was validated by Schoenfeld residuals [21]. To investigate the relationship between change in immune‐inflammation status and prognosis of ischemic stroke, we classified the immune‐inflammation levels as “mild,” “moderate,” and “severe,” based on tertiles of the index. Using these same cutoff values, the levels at the second survey were similarly categorized. With the stable status as the reference, we proceeded to compare the ORs and 95% CIs for different transition states. The covariates included sex, age, medical history, and comorbidities.

To address the missing data, multiple imputations by chained equations were applied, which was described in Supporting Information, Methods. Sensitivity analyses were conducted using the inverse probability of censoring weighting (IPCW) method [22]. In the IPCW model, predicted probabilities of complete data were estimated through a multivariable logistic regression model, and inverse probability weights based on these probabilities were applied to correct for potential biases introduced by missing data. To assess the incremental discriminative ability of CALLY, SII, and SIRI for poor outcomes, we compared *C*‐statistics and calculated the net reclassification improvement (NRI) and integrated discrimination improvement (IDI) of traditional risk models with those incorporating CALLY, SII, or SIRI [23].

To validate the relationship between changes in the immune‐inflammation state and outcomes, several sensitivity analyses were conducted: (1) the total index was the sum of two measurements. (2) change in index difference (△) was calculated by subtracting the baseline index from the second evaluation. (3) classification of participants into three groups based on changes in index values: “increase group” (≥ 10% increase from baseline, reference group), “stable group” (change between −10% and + 10%), and “decrease group” (≥ 10% decrease), with an evaluation of their association with stroke prognosis. Subgroup analyses were stratified by sex and age (middle‐aged < 70 years; elderly ≥ 70 years), and the statistical significance of the interactions was assessed using likelihood ratio tests [24]. All statistical analyses were performed using R software (version 4.1.2) with two‐tailed *p*‐values, and statistical significance was set at *p* < 0.05.

## 3 Results

### 3.1 Baseline Characteristics of the Study Population

**FIGURE 1:**
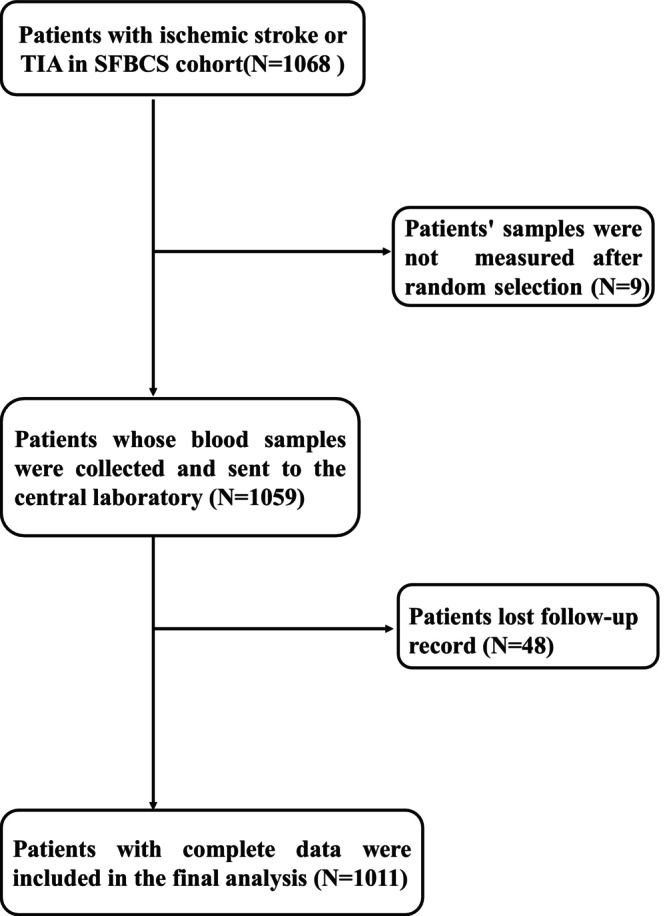
Flow chart of this study.

**FIGURE 2:**
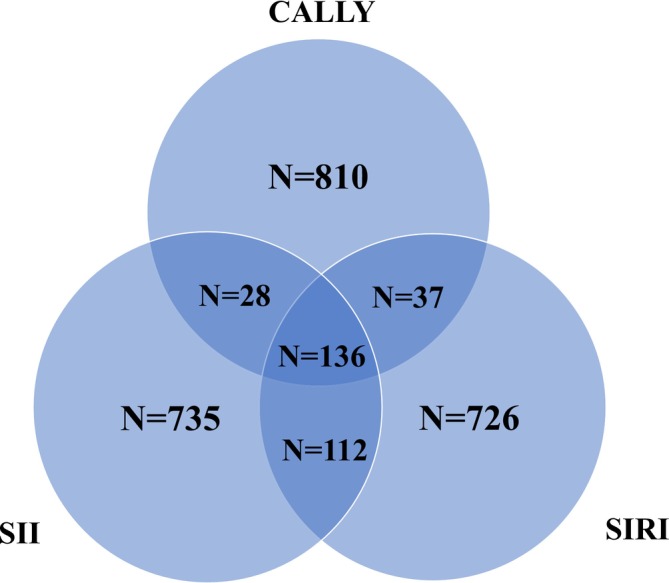
Venn diagram of inflammation risk assessed by the 3 scores.

We enrolled 1068 patients with ischemic stroke or transient ischemic attack from five centers. Individuals without inflammatory data or missing follow‐up records were excluded, resulting in a final cohort of 1011 patients (Figure 1). The cohort was stratified based on SII, SIRI, and CALLY levels, as shown in Table 1. The baseline characteristics of the included and excluded patients were similar (Table S1). At baseline, the median (interquartile range, IQR) values of the indices were 149 (36–298) for CALLY, 1.21 (0.75–2.03) for SIRI, and 625 (418–964) for SII. At the second measurement, the levels were 561 (255–1034) for CALLY, 1.47 (0.91–2.71) for SIRI, and 633 (428–1061) for SII. Patients with elevated SII and SIRI levels were generally older, whereas those with higher CALLY levels were younger. Blood glucose levels increased with higher SII and SIRI levels, but decreased with higher CALLY levels. The prevalence of hypertension was higher in patients with elevated SII and SIRI, but lower in those with higher CALLY levels. A total of 136 (13.5%) patients were identified as having a severe inflammation risk according to all three indices (Figure 2).

**TABLE 1:** Baseline characteristics of the study participants for Immune‐Inflammation status analyses.

Characteristics	SII			CALLY			SIRI		
	T1	T2	T3	T1	T2	T3	T1	T2	T3
Demographics									
Age, years	67.0 (57.0; 76.0)	66.0 (56.0; 76.0)	71.0 (60.0; 79.0)	73.0 (62.0; 81.0)	68.0 (58.0; 75.0)	64.0 (55.0; 73.0)	66.0 (55.0; 74.0)	68.0 (57.0; 78.0)	70.0 (60.0; 79.0)
Male, *n* (%)	217 (64.4)	220 (65.3)	229 (68.0)	217 (64.4)	232 (68.8)	217 (64.4)	192 (57.0)	235 (69.7)	239 (70.9)
Body mass index, kg/m^2^	24.2 (22.0; 26.1)	24.0 (21.9; 26.4)	23.7 (21.8; 25.9)	23.9 (21.5; 26.3)	23.9 (22.0; 25.9)	24.2 (22.1; 26.1)	24.1 (21.8; 26.2)	23.9 (21.9; 26.0)	24.0 (22.0; 26.1)
Fasting plasma glucose, mmol/L	5.50 (4.91; 6.83)	5.62 (4.90; 7.21)	6.07 (5.14; 7.94)	6.10 (5.13; 8.10)	5.68 (4.94; 7.08)	5.38 (4.88; 6.80)	5.51 (4.91; 6.73)	5.59 (4.88; 7.31)	6.03 (5.20; 7.83)
Total cholesterol, mmol/L	4.12 (3.47; 4.87)	4.24 (3.64; 5.04)	4.20 (3.57; 4.96)	4.06 (3.52; 4.80)	4.22 (3.64; 5.02)	4.24 (3.52; 4.98)	4.19 (3.52; 4.93)	4.19 (3.60; 4.97)	4.14 (3.53; 4.96)
Triglycerides, mmol/L	1.39 (0.99; 2.02)	1.51 (1.09; 2.06)	1.39 (1.03; 1.81)	1.33 (1.00; 1.81)	1.43 (1.04; 1.98)	1.51 (1.15; 2.10)	1.40 (1.04; 2.06)	1.44 (1.03; 1.94)	1.43 (1.05; 1.87)
LDL‐C, mmol/L	2.58 (1.94; 3.18)	2.76 (2.07; 3.44)	2.74 (2.10; 3.51)	2.61 (1.99; 3.39)	2.70 (2.07; 3.41)	2.71 (2.06; 3.31)	2.63 (1.99; 3.25)	2.71 (2.10; 3.40)	2.71 (2.02; 3.52)
HDL‐C, mmol/L	0.99 (0.83; 1.14)	0.94 (0.81; 1.14)	0.96 (0.80; 1.14)	0.93 (0.79; 1.11)	0.98 (0.83; 1.16)	0.97 (0.82; 1.14)	0.98 (0.81; 1.16)	0.97 (0.83; 1.14)	0.94 (0.79; 1.12)
Medical history, *n* (%)									
Current smoking	115 (34.1)	126 (37.4)	96 (28.5)	98 (29.1)	125 (37.1)	114 (33.8)	108 (32.0)	125 (37.1)	104 (30.9)
Current alcohol drinking	90 (26.7)	101 (30.0)	85 (25.2)	80 (23.7)	101 (30.0)	95 (28.2)	94 (27.9)	98 (29.1)	84 (24.9)
Hypertension	230 (68.2)	248 (73.6)	248 (73.6)	255 (75.7)	249 (73.9)	222 (65.9)	235 (69.7)	239 (70.9)	252 (74.8)
Diabetes	99 (29.4)	118 (35.0)	108 (32.0)	118 (35.0)	115 (34.1)	92 (27.3)	95 (28.2)	120 (35.6)	110 (32.6)
Coronary heart disease	21 (6.23)	20 (5.93)	26 (7.72)	31 (9.20)	18 (5.34)	18 (5.34)	13 (3.86)	28 (8.31)	26 (7.72)
Stroke	74 (22.0)	70 (20.8)	76 (22.6)	81 (24.0)	73 (21.7)	66 (19.6)	67 (19.9)	76 (22.6)	77 (22.8)
Hyperlipidemia	46 (13.6)	53 (15.7)	39 (11.6)	37 (11.0)	58 (17.2)	43 (12.8)	56 (16.6)	44 (13.1)	38 (11.3)
Clinical features									
Systolic BP, mmHg	153 (138; 169)	158 (140; 172)	156 (140; 170)	155 (138; 171)	157 (141; 171)	155 (140; 170)	154 (138; 170)	157 (140; 171)	156 (139; 170)
Diastolic BP, mmHg	86.0 (78.0; 95.0)	88.0 (78.0; 97.0)	86.0 (78.0; 96.0)	85.0 (77.0; 95.0)	87.0 (78.0; 96.0)	88.0 (79.0; 97.0)	86.0 (78.0; 97.0)	87.0 (78.0; 95.0)	87.0 (78.0; 97.0)
Baseline mRS score	2.00 (1.00; 3.00)	2.00 (1.00; 4.00)	3.00 (2.00; 4.00)	3.00 (2.00; 4.00)	2.00 (1.00; 4.00)	2.00 (1.00; 3.00)	2.00 (1.00; 3.00)	2.00 (1.00; 4.00)	3.00 (2.00; 4.00)
Baseline NIHSS score	3.00 (1.00; 5.00)	3.00 (2.00; 6.00)	5.00 (3.00; 13.0)	6.00 (3.00; 12.0)	3.00 (2.00; 6.00)	3.00 (1.00; 5.00)	3.00 (1.00; 5.00)	3.00 (2.00; 6.00)	6.00 (3.00; 13.0)
Ischemic stroke subtype, *n* (%)									
LAA	218 (64.7)	240 (71.2)	268 (79.5)	246 (73.0)	249 (73.9)	231 (68.5)	226 (67.1)	240 (71.2)	260 (77.2)
CE	36 (10.7)	35 (10.4)	30 (8.90)	45 (13.4)	30 (8.90)	26 (7.72)	29 (8.61)	36 (10.7)	36 (10.7)
SVO	82 (24.3)	56 (16.6)	37 (11.0)	43 (12.8)	56 (16.6)	76 (22.6)	77 (22.8)	60 (17.8)	38 (11.3)
Discharge medicine, *n* (%)									
Antiplatelet	327 (97.0)	330 (97.9)	323 (95.8)	328 (97.3)	323 (95.8)	329 (97.6)	324 (96.1)	334 (99.1)	322 (95.5)
Lipid lowering	324 (96.1)	326 (96.7)	319 (94.7)	322 (95.5)	321 (95.3)	326 (96.7)	321 (95.3)	332 (98.5)	316 (93.8)
Intravenous alteplase	52 (15.5)	49 (14.5)	60 (17.8)	65 (19.3)	45 (13.4)	51 (15.2)	53 (15.8)	50 (14.8)	58 (17.2)

Abbreviations: CALLY, C‐reactive protein‐albumin‐lymphocyte index; CE, cardioembolism; HDL‐C, high‐density lipoprotein cholesterol; IQR, interquartile range; LAA, large‐artery atherosclerosis; LDL‐C, low‐density lipoprotein cholesterol; mRS, modified Rankin Scale; NIHSS, National Institutes of Health Stroke Scale; SII, Systemic Immune Inflammation Index; SIRI, System Inflammation Response Index; SVO, small‐vessel occlusion; T, tertile.

The follow‐up periods were 3 months and 1 year. The cumulative incidence rates of poor outcomes, death, and composite vascular events at 3 months were 31.4%, 5.3%, and 2.5%, respectively. At 1 year, these rates were 27%, 9.9%, and 6.2%, respectively.

### 3.2 Association Between Immune Inflammation Index and Outcomes

**FIGURE 3:**
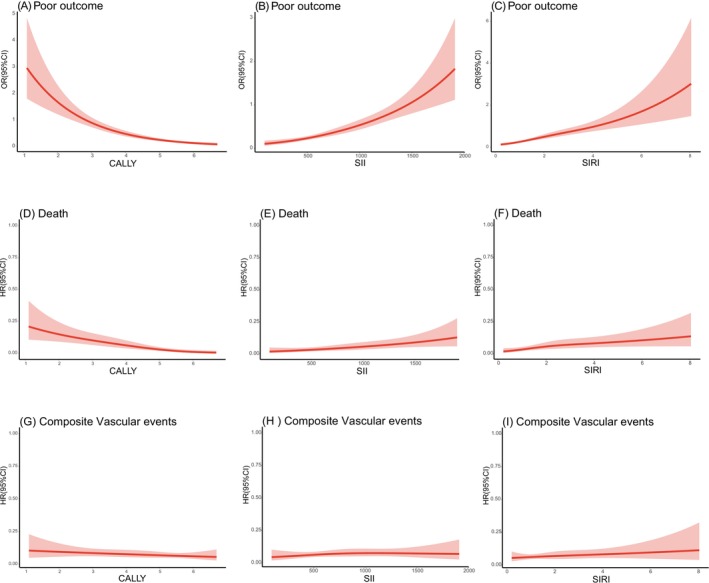
A–C: Dose–response associations between the three indices and poor outcome. D–F: Dose–response associations between the three indices and death. G–H: Dose–response associations between the three indices and composite vascular events.

After adjusting for age, sex, and other potential confounders, patients in the third quartile of CALLY demonstrated a significantly lower risk of poor outcomes than those in the first quartile (OR, 0.33 [95% CI, 0.20–0.53]; *p* < 0.001), as well as a lower risk of death (HR, 0.18 [95% CI, 0.07–0.39]; *p* < 0.001) (Table 2). No significant association was found between CALLY and the composite vascular events. A similar trend was observed at the 3‐month follow‐up (Table S2). Sensitivity analysis, which accounted for potential bias using the inverse probability of censoring weighting (IPCW) method, confirmed these associations (IPCW model in Table 2). In contrast, SII and SIRI showed different trends. Patients with high SII and SIRI levels exhibited a significantly increased risk of poor outcomes (SII: OR 2.54 [95% CI, 1.61–4.06]; *p* < 0.001; SIRI: OR 2.37 [95% CI, 1.47–3.87]; *p* < 0.001) and death (SII: HR 3.18 [95% CI, 1.54–7.13]; *p* = 0.003; SIRI: HR 4.92 [95% CI, 2.34–11.46]; *p* = 0.003). However, neither the SII nor the SIRI was directly associated with vascular events. Similar results were observed at the 3‐month follow‐up (Table S2). Additionally, restricted cubic spline analyses were performed using baseline immune‐inflammation indices to evaluate the dose–response relationship with 1‐year outcomes. These analyses showed inverse linear associations for CALLY levels and positive linear associations for SII and SIRI with poor outcomes and death (*p* for linearity < 0.001) (Figure 3; Figure S1).

**TABLE 2:** Adjusted HRs/ORs of outcomes at 1‐year according to Immune‐Inflammation Index categories.

Variable	Outcome		Events, (%)	Unadjusted model	*p*	Fully adjusted model^c^	*p*	IPCW model^a^	*p*
				HR/OR (95% CI)^b^		HR/OR (95% CI)^b^		HR/OR (95% CI)^b^	
CALLY	Poor outcome	T1	157 (12.6)	1 (Reference)		1 (Reference)		1 (Reference)	
		T2	74 (9.9)	0.32 (0.23–0.45)	< 0.001	0.55 (0.36–0.83)	0.005	0.40 (0.27–0.60)	< 0.001
		T3	42 (9.1)	0.16 (0.11–0.24)	< 0.001	0.33 (0.20–0.53)	< 0.001	0.28 (0.18–0.43)	< 0.001
	Death	T1	62 (18.4)	1 (Reference)		1 (Reference)		1 (Reference)	
		T2	25 (7.4)	0.36 (0.21–0.57)	< 0.001	0.55 (0.32–0.94)	0.031	0.46 (0.26–0.79)	0.006
		T3	7 (2.1)	0.09 (0.048–0.20)	< 0.001	0.18 (0.07–0.39)	< 0.001	0.19 (0.09–0.38)	< 0.001
	Composite vascular event	T1	26 (7.7)	1 (Reference)		1 (Reference)		1 (Reference)	
		T2	18 (5.3)	0.71 (0.38–1.31)	0.282	0.86 (0.45–1.64)	0.649	0.80 (0.42–1.52)	0.505
		T3	19 (5.6)	0.67 (0.36–1.25)	0.215	0.86 (0.44–1.69)	0.671	0.97 (0.52–1.80)	0.911
SII	Poor outcome	T1	49 (14.5)	1 (Reference)		1 (Reference)		1 (Reference)	
		T2	76 (22.6)	1.71 (1.16–2.56)	0.008	1.78 (1.11–2.87)	0.017	1.78 (1.12–2.83)	0.015
		T3	148 (43.9)	4.60 (3.19–6.72)	< 0.001	2.54 (1.61–4.06)	< 0.001	2.67 (1.73–4.17)	< 0.001
	Death	T1	10 (3.00)	1 (Reference)		1 (Reference)		1 (Reference)	
		T2	29 (8.6)	3.08 (1.52–6.74)	0.003	3.34 (1.57–7.71)	0.003	3.78 (1.82–8.38)	0.001
		T3	55 (16.3)	6.38 (3.33–13.5)	< 0.001	3.18 (1.54–7.13)	0.003	5.81 (2.95–12.40)	< 0.001
	Composite vascular event	T1	19 (5.6)	1 (Reference)		1 (Reference)		1 (Reference)	
		T2	20 (5.9)	1.06 (0.55–2.03)	0.869	1.05 (0.54–2.05)	0.880	1.14 (0.58–2.24)	0.699
		T3	24 (7.1)	1.28 (0.69–2.42)	0.432	1.17 (0.60–2.30)	0.647	1.18 (0.63–2.26)	0.606
SIRI	Poor outcome	T1	44 (13.1)	1 (Reference)		1 (Reference)		1 (Reference)	
		T2	81 (24.0)	2.11 (1.41–3.17)	< 0.001	1.89 (1.17–3.09)	0.010	2.00 (1.25–3.26)	0.004
		T3	148 (43.9)	5.21 (3.58–7.72)	< 0.001	2.37 (1.47–3.87)	< 0.001	2.44 (1.54–3.92)	< 0.001
	Death	T1	9 (2.7)	1 (Reference)		1 (Reference)		1 (Reference)	
		T2	20 (5.9)	2.30 (1.06–5.38)	0.042	2.05 (0.90–5.02)	0.097	1.89 (0.85–4.43)	0.127
		T3	65 (19.3)	8.71 (4.48–19.05)	< 0.001	4.92 (2.34–11.46)	< 0.001	6.84 (3.41–15.10)	< 0.001
	Composite vascular event	T1	24 (7.1)	1 (Reference)		1 (Reference)		1 (Reference)	
		T2	17 (5.0)	0.69 (0.36–1.31)	0.261	0.62 (0.31–1.19)	0.156	0.64 (0.33–1.23)	0.180
		T3	22 (6.5)	0.91 (0.50–1.66)	0.760	0.74 (0.40–1.44)	0.375	0.75 (0.40–1.41)	0.370

Abbreviations: CALLY, C‐reactive protein‐albumin‐lymphocyte index; SII, Systemic Immune Inflammation Index; SIRI, System Inflammation Response Index; T, tertile.

^a^ IPCW model: weighted by inverse probability for predicting complete data.

^b^ HR for death and composite vascular events; OR for poor outcome.

^c^ Fully adjusted model: adjusted for sex and age, body mass index, history of stroke, coronary heart disease, hypertension, hyperlipidemia, current smoking, drinking, pre‐stroke modified Rankin Scale, National Institutes of Health Stroke Scale score at admission, systolic blood pressure at baseline, stroke subtype.

### 3.3 Incremental Prognostic Value of Immune Inflammation Index

Table S3 illustrates the impact of incorporating the three immune‐inflammation indices into prognosis prediction along with conventional risk factors. Adding serum CALLY levels to conventional risk factors not only significantly improved discrimination ability (*C*‐statistic: 0.881 vs. 0.864; *p* = 0.011), but also enhanced risk reclassification for poor outcomes and mortality (Continuous‐NRI = 35.22%, *p* < 0.001; 1‐year IDI = 0.24%, *p* < 0.001). However, it did not improve the discrimination of composite vascular events. Incorporating serum SII levels alongside conventional risk factors did not enhance discrimination, but significantly improved risk reclassification for poor outcomes (Continuous‐NRI = 26.44%, *p* < 0.001; IDI = 0.07%, *p* = 0.003) and mortality (Continuous‐NRI = 23.07%, *p* = 0.033; IDI = 0.09%, *p* = 0.042). However, the SIRI did not demonstrate any significant improvement across any of the indices The same trend was obvious in 3 months (Table S4).

### 3.4 Association of Changes in Immune‐Inflammation Status With Poor Prognosis

Table S5 shows the number and percentage of participants with changes in the immune‐inflammation status. Among the baseline participants, 18 individuals (5.4%) in the CALLY index, 116 individuals (34.4%) in the SII, and 174 individuals (51.6%) in the SIRI progressed to moderate–severe states. Among participants with severe status at baseline, 238 patients (70.6%), 110 patients (32.7%), and 102 patients (30.3%) showed improvement in their immune‐inflammation status.

Table 3 demonstrates the association between changes in immune‐inflammation status and cerebrovascular outcomes. Compared to participants with stable mild inflammation, those who progressed to severe status had significantly higher risks of poor outcomes. In contrast, participants who recovered to mild status from severe inflammation had a significantly lower risk of poor outcomes. However, changes in immune‐inflammation status indices did not appear to have significant associations with vascular event outcomes.

**TABLE 3:** Association of changes in Immune‐Inflammation Index with stroke prognosis.

	CALLY				SII				SIRI			
	HR/OR (95% CI)	*p*	Adjusted HR/OR (95% CI)	*p*	HR/OR (95% CI)	*p*	Adjusted HR/OR (95% CI)	*p*	HR/OR (95% CI)	*p*	Adjusted HR/OR (95% CI)	*p*
Poor outcome												
Stable mild	1 (reference)		1 (reference)		1 (reference)		1 (reference)		1 (reference)		1 (reference)	
Mild to moderate	5.82 (1.45–20.52)	0.007	2.25 (1.22–15.11)	0.003	4.67 (2.25–9.71)	< 0.001	1.49 (1.77–1.20)	0.002	2.44 (1.19–4.99)	0.014	3.19 (1.30–8.02)	0.012
Mild to severe	33.93 (9.74–58.05)	< 0.001	14.2 (2.31–64.27)	0.008	3.46 (1.59–7.42)	< 0.001	1.27 (1.00–7.10)	0.050	3.89 (1.82–8.24)	< 0.001	4.10 (1.61–10.71)	0.003
Stable moderate	(reference)		(reference)	< 0.001	(reference)		(reference)		(reference)		(reference)	
Moderate to mild	0.3 (0.15–0.71)	0.004	0.3 (0.12–0.73)	0.017	0.4 (0.16–0.91)	0.040	0.3 (0.11–0.89)	0.039	0.2 (0.09–0.67)	0.009	0.2 (0.08–0.85)	0.037
Moderate to severe	3.8 (1.30–12.49)	0.018	2.1 (1.08–10.31)	0.033	3.3 (1.81–6.15)	< 0.001	2.3 (0.67–3.64)	0.043	2.1 (1.26–3.76)	0.006	1.9 (1.02–3.91)	0.044
Stable severe					(reference)		(reference)		(reference)		(reference)	
Severe to moderate	0.4 (0.21–0.78)	0.007	0.4 (0.19–0.86)	0.02	0.2 (0.15–0.51)	< 0.001	0.3 (0.14–0.66)	0.003	0.3 (0.21–0.67)	0.001	0.6 (0.30–1.43)	0.299
Severe to mild	0.1 (0.06–0.18)	< 0.001	0.2 (0.10–0.38)	< 0.001	0.3 (0.13–0.62)	0.002	0.6 (0.24–1.70)	0.405	0.1 (0.06–0.38)	< 0.001	0.2 (0.07–0.76)	0.022
Death												
Stable mild	(reference)		(reference)		(reference)		(reference)		(reference)		(reference)	
Mild to moderate	7.7 (0.38–58.57)	0.079	6.2 (0.51–21.33)	0.814	3.8 (0.70–21.53)	0.102	1.0 (0.37–57.76)	0.271	2.4 (0.57–10.67)	0.209	3.8 (0.67–24.59)	0.130
Mild to severe	14.0 (1.81–80.59)	0.004	9.3 (1.92–31.84)	0.041	8.5 (2.18–41.58)	0.003	1.0 (1.59–150.94)	0.029	4.8 (1.25–20.33)	0.022	5.2 (1.02–30.56)	0.048
Stable moderate	0.1 (0.08–0.23)		(reference)		(reference)	< 0.001	(reference)	0.005	(reference)		(reference)	
Moderate to mild	0.1 (0.05–0.55)	0.002	0.0 (0.01–0.31)	0.001	0.2 (0.01–1.03)	0.123	0.2 (0.01–1.27)	0.168	0.6 (0.09–2.79)	0.571	0.5 (0.07–2.73)	0.497
Moderate to severe	7.8 (2.34–29.79)	0.001	10.8 (1.95–78.56)	0.010	3.9 (1.71–9.13)	0.001	3.9 (1.44–11.21)	0.008	2.9 (1.13–8.47)	0.034	2.7 (1.01–8.66)	0.049
Stable severe	(reference)		(reference)		(reference)		(reference)		(reference)		(reference)	
Severe to moderate	0.2 (0.12–0.54)	< 0.001	0.2 (0.11–0.55)	< 0.001	0.2 (0.16–0.80)	0.019	0.2 (0.08–0.81)	0.031	0.3 (0.15–0.80)	0.019	0.5 (0.20–1.49)	0.280
Severe to mild	0.0 (0.02–0.15)	< 0.001	0.0 (0.03–0.20)	< 0.001	0.0 (0.03–0.61)	0.02	0.1 (0.01–0.93)	0.102	0.1 (0.03–0.61)	0.020	0.3 (0.06–1.41)	0.204
Composite vascular event												
Stable mild	(reference)		(reference)		(reference)		(reference)		(reference)		(reference)	
Mild to moderate	1.8 (0.10–10.63)	0.567	1.3 (0.06–9.48)	0.795	1.2 (0.34–3.80)	0.692	0.7 (0.15–2.56)	0.608	4.1 (1.59–11.78)	0.004	4.2 (1.47–12.88)	0.008
Mild to severe	3.3 (0.49–14.00)	0.133	2.3 (0.29–13.17)	0.360	1.3 (0.36–3.95)	0.647	1.1 (0.28–3.84)	0.865	2.1 (0.55–7.41)	0.236	2.0 (0.47–7.61)	0.315
Stable moderate	(reference)		(reference)		(reference)		(reference)		(reference)		(reference)	
Moderate to mild	0.9 (0.23–6.86)	0.934	0.3 (0.16–0.71)	0.975	0.4 (0.07–1.85)	0.351	0.4 (0.06–1.86)	0.332	0.3 (0.05–1.43)	0.200	0.4 (0.06–1.85)	0.313
Moderate to severe	0.7 (0.03–10.43)	0.819	0.2 (0.10–0.39)	0.824	1.5 (0.07–1.85)	0.434	1.4 (0.37–4.99)	0.569	0.8 (0.28–2.14)	0.657	0.7 (0.26–2.22)	0.639
Stable severe	(reference)		(reference)		(reference)		(reference)		(reference)		(reference)	
Severe to moderate	1.9 (0.63–6.04)	0.252	1.7 (0.56–5.97)	0.325	0.9 (0.30–2.46)	0.893	0.9 (0.28–2.62)	0.895	1.3 (0.46–3.48)	0.567	1.3 (0.41–3.75)	0.639
Severe to mild	1.2 (0.46–3.59)	0.706	1.6 (0.56–5.51)	0.377	1.0 (0.22–3.15)	0.996	1.1 (0.23–4.02)	0.869	0.7 (0.12–2.89)	0.731	0.8 (0.12–3.68)	0.313

Abbreviations: CALLY, C‐reactive protein‐albumin‐lymphocyte index; HR, hazard ratio; OR, odds ratio; SII, Systemic Immune Inflammation Index; SIRI, System Inflammation Response Index; T, tertile.

### 3.5 Sensitivity Analyses

We evaluated the relationship between the total CALLY, SII, and SIRI levels and outcomes (Tables S6 and S7). After adjusting for confounders, participants in the upper third quartile of total SII and SIRI showed a significantly higher risk of poor outcomes during both short‐ and long‐term follow‐ups than those in the lower third quartile. In contrast, CALLY demonstrated the opposite result. Participants in the middle tertile of the total index also exhibited a significantly higher risk of poor outcomes. The same trend was observed for mortality outcomes (all *p* < 0.001). However, for participants in the middle tertile of the total index, significantly increased risks of death were only observed for CALLY but not for SII and SIRI.

Tables S8 and S9 shows the association between changes in the △CALLY, △SII, and △SIRI levels and the risk of outcomes. Participants in the upper tertile of △CALLY, △SII, and △SIRI exhibited significantly higher risks of poor outcomes compared to those in the lower tertile for CALLY and SII (OR, 0.62 [95% CI, 0.40–0.97] for CALLY; OR, 1.61 [95% CI, 1.06–2.47] for SII), but not for SIRI. For mortality outcomes, only CALLY and SIRI showed consistent results (CALLY: HR, 0.35 [95% CI, 0.18–0.63], *p* < 0.001; SIRI: HR, 2.08 [95% CI, 1.23–3.59], *p* < 0.001). No significant changes were observed during the 3‐month follow‐up. No statistically significant difference was found with respect to the risk of vascular events. The risk of adverse prognosis was higher in the “increase group” for SII and SIRI compared to the “decrease group,” whereas the opposite was observed for CALLY (Tables S10 and S11). In stratified analyses, similar results were found for both males and females (Table S12). The results were also consistent among the middle‐aged and older participants (Table S13).

## 4 Discussion

This prospective cohort study examined the relationship between baseline changes in inflammatory status and stroke prognosis. We found that elevated baseline CALLY levels were associated with a reduced risk of both short‐ and long‐term poor outcomes in patients with ischemic stroke. In contrast, higher SII and SIRI levels were linked to an increased risk of poor outcomes over both timeframes. Incorporating these indices alongside conventional risk factors improved the predictive accuracy, with CALLY providing the most significant improvement in predictive accuracy, as demonstrated by the NRI and IDI. Patients with severe inflammation are at a higher risk of poor outcomes. Specifically, those who progressed to a severe inflammatory state were at a higher risk than those with stable mild inflammation. Conversely, patients with severe inflammation who recovered to a mild inflammatory state had a lower risk than those with stable mild inflammation. Moreover, higher total indices and their increase were associated with an elevated risk of poor outcomes.

The immune system plays a crucial role in the pathogenesis of ischemic stroke. Neuronal damage triggers secondary inflammatory responses that exacerbate brain injury and neurological deficits [25]. Previous studies have demonstrated a strong association between immune‐inflammation status and post‐stroke prognosis [11, 26, 27]. For example, a mediation analysis suggested that inflammation mediates insulin resistance, contributing to poor stroke outcomes [28]. A large cohort study in China found that inflammation was linked to poor functional outcomes 1 year after acute ischemic stroke, although this association was observed only in patients without chronic kidney disease (CKD) [29]. Our study aligns with these findings, showing that elevated immune‐inflammation markers are associated with both short‐ and long‐term poor outcomes [30]. This association may be mediated by underlying mechanisms activated after stroke, such as ischemic cascades and vascular damage, which lead to the infiltration of inflammatory cells into brain tissue [31]. Pro‐inflammatory signals activate neutrophils, monocytes, and other immune cells, exacerbating brain tissue damage. This leads to the production of reactive oxygen species, exudation, and extracellular traps, all of which contribute to stroke severity [32, 33, 34]. Notably, neutrophils are among the first immune cells to respond to ischemic brain injury, and their effects on systemic inflammation and the blood–brain barrier are correlated with stroke severity [35]. Monocytes also activate platelets, forming platelet‐monocyte aggregates that promote inflammation [36, 37]. This pathophysiological process highlights how the SII and SIRI reflect the systemic immune‐inflammation status and predict stroke outcomes. Furthermore, compared to SII, SIRI, the CALLY demonstrated higher predictive accuracy in our cohort. This likely stems from the CALLY index's incorporation of albumin, a multifunctional protein that regulates colloid osmotic pressure and modulates critical biological processes including thrombosis suppression, platelet aggregation inhibition, antioxidant activity, and anti‐inflammatory responses [38, 39].

Beyond the baseline status, our study also investigated dynamic changes in the immune‐inflammation state and their impact on stroke prognosis, an area that is relatively underexplored. We found that patients who progressed to a severe inflammatory state had an increased risk of poor outcomes compared with those with stable mild inflammation, emphasizing the detrimental impact of inflammation progression on stroke recovery. Conversely, patients who recovered from severe inflammation to a mild inflammatory state had a reduced risk of poor outcomes. The dynamic nature of immune severity was further assessed through total inflammation indices and changes in groupings, with high indices and significant changes associated with a poor prognosis. These findings suggest that interventions aimed at reversing inflammation might offer potential therapeutic benefits. However, a multicenter, double‐blind, randomized, placebo‐controlled trial in China found that anti‐inflammatory treatment did not reduce the risk of stroke recurrence [40]. Potential explanations include inadequate treatment duration and lack of longitudinal inflammation monitoring. Nevertheless, subgroup analyses indicated that low‐dose colchicine treatment may benefit certain patients, suggesting that anti‐inflammatory treatment could be effective in specific subgroups. Early dynamic immune‐inflammation assessment may help identify candidates for early immunomodulatory therapy, particularly in those exhibiting inflammatory progression. Further clinical trials are needed to explore optimal treatment protocols.

This study has several strengths. It is the first to investigate the association between changes in immune‐inflammation status and the risk of poor outcomes after stroke. We used three novel indicators to characterize immune‐inflammation conditions post‐stroke, and our results were consistent across all three groups. Sensitivity analyses further confirmed the robustness of our findings. However, this study has some limitations. First, the assessment of the dynamic immune‐inflammation status was based on only two pre‐admission examinations, which may not fully capture long‐term changes. Future studies should extend the evaluation period to achieve higher accuracy. Second, although we adjusted for multiple confounding factors, other unmeasured variables such as genetic susceptibility may have influenced the results. Sensitivity analyses using the IPCW method for missing data confirmed the consistency of the primary findings. Third, due to the lack of standardized classification criteria, we used percentiles to represent immune‐inflammation status, which may have introduced some bias. Nonetheless, the overall trend was consistent. Finally, the differences in stroke subtypes among Chinese patients may limit the generalizability of our findings to other populations.

## 5 Conclusion

CALLY demonstrated superior predictive ability for stroke outcomes compared with SII and SIRI. Varying changes in immune‐inflammation states are associated with different risks of poor prognosis in stroke patients. Further research is needed to develop targeted preventive strategies, providing a theoretical foundation for early intervention in patients with acute ischemic stroke.

## Author Contributions

Keyang Chen, and Beilei Hu contributed to the conception and design of the study; Wenting Huang, Yitian Liu, Xuan Chen, Qian Shen, Hanyu Cai, Jing Sun, Yan Li, Yungang Cao, contributed to the acquisition and analysis of data; Songfang Chen and Wenting Huang contributed to drafting the text or preparing the figures.

## Ethics Statement

The study was approved by the Ethics Committee of Wenzhou Medical University and institutional review boards (no. YJ‐2021‐K‐73‐02).

## Conflicts of Interest

The authors declare no conflicts of interest.

## Supporting information


**Data S1:** Supporting Information.

## Data Availability

The supporting data for the results of the study can be obtained from the corresponding authors.
