# Supplementary material for: Changes in Immune‐Inflammation Status and Acute Ischemic Stroke Prognosis in Prospective Cohort
Source: Ann Clin Transl Neurol. 2025 Nov 24;13(4):767–79. doi: 10.1002/acn3.70252 (PMC13071089; doi:10.1002/acn3.70252)
Supplement: Supplementary file 1 — Data S1: Supporting Information. [file ACN3-13-767-s001.docx]

**Supplemental Methods**

**Multiple imputation**

The missing data of covariates were imputed using the multiple imputation with chained equation. In our cohort, we only imputed the covariates in which the missing rates were less than 20% . All eligible covariates were imputed using one imputation model which included the sex and age, body mass index, history of stroke, coronary heart disease, hypertension, hyperlipidemia, current smoking, drinking, pre‐stroke modified Rankin Scale, National Institutes of Health Stroke Scale score, systolic blood pressure, stroke subtype. We performed 10 imputations and generated 10 imputed datasets. Effect estimates were computed separately for each of the 10 datasets, and then combined according to Rubin’s rules. The multiple imputation was conducted using the R package "mice"^1^.

**Reference**

1. White IR, Royston P, Wood AM. Multiple imputation using chained equations: Issues and guidance for practice. *Stat Med*. 2011;30:377-399.doi:10.1002/sim.4067:

Figure S1. Dose-response association of immune-inflammation index with 3- months outcomes.

**
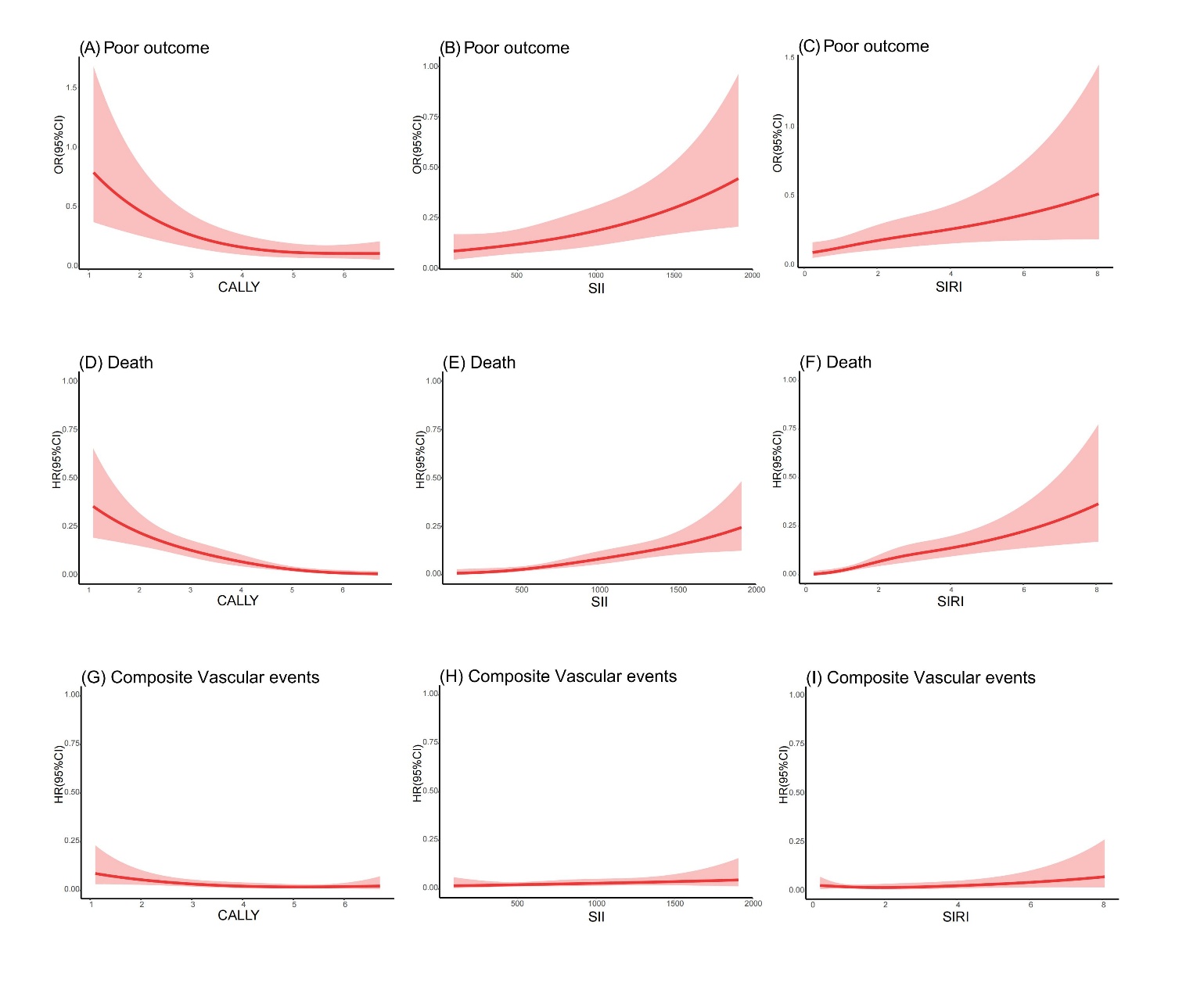
**

**Table S1. Baseline clinical characteristics between study population and excluded population**

|  | Study population | Excluded population | P-value | |
| --- | --- | --- | --- | --- |
| **Demographics** |  |  | |  |
| Age, y | 67.2 (12.9) | 66.9(13.0) | 0.32 | |
| Male, n (%) | 666 (65.9) | 39(68.4) | 0.80 | |
| Current cigarette smoking, n (%) | 337 (33.3) | 18(31.6) | 0.90 | |
| Current alcohol drinking, n (%) | 276 (27.3) | 15(26.3) | 0.99 | |
| **Medical history** |  |  |  | |
| Hypertension, n (%) | 726 (71.8) | 42(73.7) | 0.88 | |
| Diabetes, n (%) | 325 (32.2) | 19(33.3) | 0.93 | |
| Coronary heart disease, n (%) | 67 (6.6) | 5(7.2) | 0.72 | |
| Family history of stroke, n (%) | 220 (21.8) | 14(24.6) | 0.74 | |
| Hyperlipidemia, n (%) | 86(8.5) | 4(7.0) | 0.88 | |
| **Clinical features** |  |  |  | |
| Systolic BP at baseline, mmHg | 156 (139-171) | 156 (137-169) | 0.82 | |
| Body mass index, kg/m^2^ | 24.0 (21.9-26.0) | 24.2 (22.3-26.7) | 0.33 | |
| Baseline NIHSS score | 4.00 (2.00-7.00) | 4.00 (2.00-7.00) | 0.07 | |
| Fasting plasma glucose, mmol/L | 5.68 (4.94-7.40) | 5.68 (4.98-7.66) | <0.001 | |
| Total cholesterol, mmol/L | 4.17 (3.56-4.96) | 4.16 (3.53-4.97) | <0.001 | |
| Triglycerides, mmol/L | 1.43 (1.04-1.96) | 1.43 (1.04-1.95) | 0.187 | |
| LDL-C, mmol/L | 2.68 (2.05-3.39) | 2.67 (2.05-3.32) | <0.001 | |
| HDL-C, mmol/L | 0.96 (0.81-1.14) | 0.97 (0.82-1.14) | <0.001 | |
| **Ischemic stroke subtype, n (%)** |  |  |  | |
| Thrombotic | 726 (71.8) | 42(73.7) | 0.88 | |
| Embolic | 101 (10.0) | 6(8.8) | 0.94 | |
| Lacunar | 175 (17.3) | 10(15.8) | 0.91 | |
| Continuous data are presented as median (interquartile range, IQR), and categorical variables are presented as %. HDL-C: high-density lipoprotein cholesterol; LDL-C, low-density lipoprotein cholesterol; NIHSS, National Institutes of Health Stroke Scale; | | | | |

**Table S2. Adjusted HRs/ORs of Outcomes at 3 Months According to Immune-Inflammation Index Categories**

| Variable |  |  | Events, n (%) | Unadjusted model | P value | Fully adjusted model* | P value | IPCW model† | P value |
| --- | --- | --- | --- | --- | --- | --- | --- | --- | --- |
|  |  |  |  | HR/OR (95% CI)‡ |  | HR/OR (95% CI)‡ |  | HR/OR (95% CI)‡ |  |
| CALLY | Poor outcome[δ](https://www.ahajournals.org/reader/content/193898496ed/10.1161/JAHA.124.036721/format/epub/EPUB/xhtml/index.xhtml?hmac=1740987712-bHV2el4kiOkPbVQgGhypE%2BtLNbRT4W7xOwp8kqwW5Y8%3D#jah310364-note-0010) | T1 | 171(8.3) | 1 (Reference) |  | 1 (Reference) |  | 1 (Reference) |  |
|  |  | T2 | 91(5.9) | 0.36(0.26-0.49) | 0.302 | 0.59(0.39-0.88) | 0.010 | 0.42(0.28-0.62) | <0.001 |
|  |  | T3 | 55(4.3) | 0.19(0.13-0.27) | 0.072 | 0.36(0.23-0.56) | <0.001 | 0.31(0.21-0.47) | <0.001 |
|  | Death | T1 | 34(10.1) | 1 (Reference) |  | 1 (Reference) |  | 1 (Reference) |  |
|  |  | T2 | 15(4.5) | 0.42(0.22-0.76) | 0.006 | 0.85(0.41-1.70) | 0.648 | 0.33(0.17-0.58) | <0.001 |
|  |  | T3 | 5(1.5) | 0.13(0.05-0.32) | <0.001 | 0.38(0.12-0.98) | 0.042 | 0.23(0.11-0.43) | <0.001 |
|  | Composite Vascular Event | T1 | 14(4.2) | 1 (Reference) |  | 1 (Reference) |  | 1 (Reference) |  |
|  |  | T2 | 2(0.6) | 0.14(0.02-0.50) | 0.009 | 0.15(0.02-0.58) | 0.016 | 0.14(0.02-0.54) | 0.014 |
|  |  | T3 | 9(2.7) | 0.63(0.26-1.46) | 0.293 | 0.83(0.32-2.08) | 0.699 | 0.98(0.42-2.26) | 0.960 |
| SII | Poor outcome | T1 | 61(18.1) | 1 (Reference) |  | 1 (Reference) |  | 1 (Reference) |  |
|  |  | T2 | 87(25.8) | 1.57(1.09-2.29) | 0.016 | 1.53(0.98-2.39) | 0.06 | 4.87(1.60-18.41) | 0.009 |
|  |  | T3 | 169(29.2) | 4.55(3.22-6.49) | <0.001 | 2.60(1.68-4.04) | <0.001 | 9.53(3.50-33.65) | <0.001 |
|  | Death | T1 | 4(1.2) | 1 (Reference) |  | 1 (Reference) |  | 1 (Reference) |  |
|  |  | T2 | 13(3.9) | 3.34(1.17-11.96) | 0.036 | 4.49(1.45-17.56) | 0.016 | 3.57(1.29-11.5) | 0.021 |
|  |  | T3 | 37(11.0) | 10.27(4.06-34.59) | <0.001 | 5.22(1.85-19.14) | 0.005 | 6.35(2.55-19.2) | <0.001 |
|  | Composite Vascular Event | T1 | 6(1.8) | 1 (Reference) |  | 1 (Reference) |  | 1 (Reference) |  |
|  |  | T2 | 7(2.1) | 2.09(1.44-3.05) | <0.001 | 1.88(1.20-2.98) | 0.006 | 2.03(1.29-3.20) | 0.002 |
|  |  | T3 | 12(3.6) | 5.15(3.61-7.44) | <0.001 | 2.48(1.58-3.93) | <0.001 | 2.82(1.83-4.42) | <0.001 |
| SIRI | Poor outcome | T1 | 54(16.0) | 1 (Reference) |  | 1 (Reference) |  | 1 (Reference) |  |
|  |  | T2 | 96(28.5) | 1.17(0.38-3.67) | 0.78 | 1.00(0.32-3.20) | 0.998 | 1.20(0.38-3.97) | 0.752 |
|  |  | T3 | 167(49.6) | 2.04(0.78-5.91) | 0.16 | 1.54(0.55-4.75) | 0.423 | 1.45(0.51-4.51) | 0.495 |
|  | Death | T1 | 5(1.5) | 1 (Reference) |  | 1 (Reference) |  | 1 (Reference) |  |
|  |  | T2 | 5(1.5) | 1.00(0.28-3.63) | 0.59 | 0.83(0.22-3.22) | 0.785 | 0.90(0.25-3.25) | 0.874 |
|  |  | T3 | 44(13.1) | 9.54(4.28-12.93) | <0.001 | 4.90(1.86-15.73) | 0.003 | 8.28(3.40-24.46) | <0.001 |
|  | Composite Vascular Event t | T1 | 11(3.3) | 1 (Reference) |  | 1 (Reference) |  | 1 (Reference) |  |
|  |  | T2 | 6(1.8) | 0.54(0.18-1.43) | 0.226 | 0.46(0.15-1.28) | 0.148 | 0.35(0.11-0.97) | 0.051 |
|  |  | T3 | 8(2.4) | 0.72(0.28-1.80) | 0.487 | 0.44(0.15-1.24) | 0.130 | 0.31(0.10-1.18) | 0.054 |
| CALLY, C-reactive protein-albumin-lymphocyte index; SII, Systemic Immune Inflammation Index; SIRI, System Inflammation Response Index; T, tertile; †: IPCW model: weighted by inverse probability for predicting complete data. ‡:HR for death and composite vascular events; OR for poor outcome. *Fully adjusted model: adjusted for sex and age, body mass index, history of stroke, coronary heart disease, hypertension, hyperlipidemia, current smoking, drinking, pre‐stroke modified Rankin Scale, National Institutes of Health Stroke Scale score at admission, systolic blood pressure at baseline, stroke subtype. | | | | | | | | | |

**Table S3. Reclassification and Discrimination Statistics for 1-year Clinical Outcomes by Immune-Inflammation Index**

|  | C-statistic |  | Continuous-NRI |  | IDI |  |
| --- | --- | --- | --- | --- | --- | --- |
|  | Estimate (95% CI), % | P value | Estimate  (95% CI), % | P value | Estimate  (95% CI), % | P value |
| Poor outcome |  |  |  |  |  |  |
| Conventional model | 0.864(0.838-0.890) | 1 (Reference) |  | 1 (Reference) |  | 1 (Reference) |
| Conventional model+CALLY | 0.881(0.854-0.907) | 0.011 | 35.22(21.52-48.93) | <0.001 | 0.24(0.12-0.36) | <0.001 |
| Conventional model+SII | 0.870(0.845-0.895) | 0.143 | 26.44(12.66-40.23) | <0.001 | 0.07(0.02-0.12) | 0.003 |
| Conventional model+SIRI | 0.867(0.842-0.892) | 0.282 | -4.47(-17.77-8.84) | 0.511 | 0.04(0.00-0.08) | 0.051 |
| Death |  |  |  |  |  |  |
| Conventional model | 0.875(0.859-0.891) | 1 (Reference) |  | 1 (Reference) |  | 1 (Reference) |
| Conventional mode+CALLY | 0.912(0.896-0.928) | 0.039 | 47.29(26.65-67.92) | <0.001 | 0.41(0.18-0.64) | <0.001 |
| Conventional model+SII | 0.883(0.867-0.899) | 0.235 | 23.07(1.91-44.23) | 0.033 | 0.09(0.00-0.18) | 0.042 |
| Conventional model+SIRI | 0.878(0.862-0.894) | 0.500 | 17.39(-3.65-38.43) | 0.105 | 0.09(0.00-0.18) | 0.055 |
| Composite Vascular events |  |  |  |  |  |  |
| Conventional model | 0.661(0.626-0.696) | 1 (Reference) |  | 1 (Reference) |  | 1 (Reference) |
| Conventional model+CALLY | 0.662(0.627-0.697) | 0.934 | 0.27(-24.97-25.51) | 0.983 | 0.02(-0.05-0.07) | 0.813 |
| Conventional model+SII | 0.665(0.631-0.699) | 0.346 | -3.11(-28.23-22.00) | 0.808 | -0.02(-0.11-0.07) | 0.693 |
| Conventional model+SIRI | 0.665(0.631-0.699) | 0.370 | -3.38(-27.44-20.69) | 0.783 | -0.03(-0.18-0.12) | 0.686 |
| Conventional model included sex and age, body mass index, history of stroke, coronary heart disease, hypertension, hyperlipidemia, current smoking, drinking, pre-stroke modified Rankin Scale, NIHSS score at admission, stroke subtype; IDI, integrated discrimination improvement; mRS, modified Rankin Scale; NIHSS, National Institutes of Health Stroke Scale; and NRI, net reclassification index. | | | | | | |

**Table S4. Reclassification and Discrimination Statistics for 3-Month Clinical Outcomes by Immune-Inflammation Index**

|  | C-statistic |  | Continuous-NRI |  | IDI |  |
| --- | --- | --- | --- | --- | --- | --- |
|  | Estimate (95% CI), % | P value | Estimate (95% CI), % | P value | Estimate (95% CI), % | P value |
| Poor outcome† |  |  |  |  |  |  |
| Conventional model | 0.851(0.826-0.876) | 1 (Reference) |  | 1 (Reference) |  | 1 (Reference) |
| Conventional model+CALLY | 0.870(0.846-0.894) | 0.004 | 45.35(32.39-58.331) | <0.001 | 2.63(1.45-3.81) | <0.001 |
| Conventional model+SII | 0.858(0.833-0.883) | 0.151 | 34.13(21.03-47.22) | <0.001 | 1.59(0.72-2.45) | <0.001 |
| Conventional model+SIRI | 0.856(0.831-0.881) | 0.140 | 21.55(8.73-34.38) | <0.001 | 1.24(0.42-2.06) | 0.003 |
| Death |  |  |  |  |  |  |
| Conventional model | 0.880(0.856-0.904) | 1 (Reference) |  | 1 (Reference) |  | 1 (Reference) |
| Conventional model+CALLY | 0.916(0.892-0.940) | 0.033 | 56.51(29.51-83.51) | <0.001 | 2.49(-0.79-5.76) | 0.137 |
| Conventional model+SII | 0.884(0.860-0.908) | 0.514 | 48.78 (21.73-75.82) | <0.001 | 1.14(-0.59-2.87) | <0.001 |
| Conventional model+SIRI | 0.882(0.858-0.906) | 0.778 | 27.665(0.51-54.8) | 0.046 | 1.44(-0.75-3.63) | 0.198 |
| Composite Vascular events |  |  |  |  |  |  |
| Conventional model | 0.717(0.671-0.763) | 1 (Reference) |  | 1 (Reference) |  | 1 (Reference) |
| Conventional model+CALLY | 0.721(0.675-0.767) | 0.808 | 32.28(-7.11 -71.68) | 0.108 | 0.24(-0.16-0.64) | 0.236 |
| Conventional model+SII | 0.719(0.673-0.765) | 0.809 | 6.46(-32.94-45.86) | 0.748 | 0.07(-0.13-0.27) | 0.482 |
| Conventional model+SIRI | 0.717(0.671-0.763) | 0.967 | -26.24(-60.26-7.78) | 0.131 | 0.04(-0.11-0.19) | 0.585 |
| Conventional model included sex and age, body mass index, history of stroke, coronary heart disease, hypertension, hyperlipidemia, current smoking, drinking, pre-stroke modified Rankin Scale, NIHSS score at admission, stroke subtype; IDI, integrated discrimination improvement; mRS, modified Rankin Scale; NIHSS, National Institutes of Health Stroke Scale; and NRI, net reclassification index. | | | | | | |

**Table S5. Number and percentage of the changes in Immune-Inflammation status**

| Baseline | The second survey | CALLY | SII | SIRI |
| --- | --- | --- | --- | --- |
|  | Mild | 319(94.6) | 221(65.6) | 163(48.4) |
| Mild | Moderate | 10(3.0) | 59(17.5) | 105(31.2) |
|  | Severe | 8(2.4) | 57(16.9) | 69(20.4) |
|  | Mild | 279(82.8) | 74(22.0) | 61(18.1) |
| Moderate | Moderate | 35(10.4) | 202(59.9) | 146(43.3) |
|  | Severe | 23(6.8) | 61(18.1) | 130(38.6) |
|  | Mild | 165(49.0) | 40(11.9) | 29(8.6) |
| Severe | Moderate | 73(21.6) | 70(20.8) | 73(21.7) |
|  | Severe | 99(29.4) | 227(67.3) | 235(69.3) |

**Table S6. Association of total in Immune-Inflammation Index with stroke prognosis in 1-year**

|  | CALLY | | | | | SII | | | | | SIRI | | | | |
| --- | --- | --- | --- | --- | --- | --- | --- | --- | --- | --- | --- | --- | --- | --- | --- |
| Poor outcome | | | | | | | | | | | | | | | |
|  | Events, (%) | HR/OR  (95% CI) | P | HR/OR  (95% CI) | P | Events, (%) | HR/OR  (95% CI) | P | HR/OR  (95% CI) | P | Events, (%) | HR/OR  (95% CI) | P | HR/OR  (95% CI) | P |
| TotalT1 | 180/337 | 1(reference) |  | 1(reference) |  | 45/337 | 1(reference) |  | 1(reference) |  | 44/337 | 1(reference) |  | 1(reference) |  |
| TotalT2 | 59/337 | 0.19(0.13-0.26) | <0.001 | 0.34(0.22-0.52) | <0.001 | 74/337 | 1.83(1.22-2.76) | 0.004 | 1.69(1.05-2.75) | 0.032 | 80/337 | 2.07(1.39-3.13) | <0.001 | 1.95(1.20-3.21) | 0.007 |
| TotalT3 | 34/337 | 0.10(0.06-0.15) | <0.001 | 0.23(0.14-0.36) | <0.001 | 154/337 | 5.46(1.29-2.51) | <0.001 | 2.60(1.06-2.47) | <0.001 | 149/337 | 5.28(3.63-7.81) | <0.001 | 2.69(1.67-4.40) | <0.001 |
| Death | | | | | | | | | | | | | | | |
| TotalT1 | 81/337 | 1(reference) |  | 1(reference) |  | 13/337 | 1(reference) |  | 1(reference) |  | 11/337 | 1(reference) |  | 1(reference) |  |
| TotalT2 | 11/337 | 0.11(0.05-0.20) | <0.001 | 0.16(0.08-0.31) | <0.001 | 19/337 | 0.57(0.31-1.02) | 0.061 | 1.49(0.73-3.13) | 0.28 | 19/337 | 1.77(0.84-3.90) | 0.14 | 1.77(0.73-3.69) | 0.250 |
| TotalT3 | 2/337 | 0.02(0.003-0.06) | <0.001 | 0.04(0.01-0.12) | <0.001 | 62/337 | 1.39(0.86-2.28) | 0.179 | 5.62(3.12-10.88) | <0.001 | 64/337 | 6.95(3.74-14.16) | <0.001 | 6.95(2.00-8.69) | <0.001 |
| Composite Vascular events | | | | | | | | | | | | | | | |
| TotalT1 | 28/337 | 1(reference) |  | 1(reference) |  | 19/337 |  |  |  |  | 19/337 | 1(reference) |  | 1(reference) |  |
| TotalT2 | 17/337 | 0.59(0.31-1.08) | 0.093 | 0.71(0.36-1.37) | 0.308 | 21/337 | 1.11(0.59-2.13) | 0.744 | 1.13(0.59-2.20) | 0.706 | 23/337 | 1.23(0.66-2.32) | 0.524 | 1.21(0.63-2.34) | 0.568 |
| TotalT3 | 18/337 | 0.62(0.33-1.14) | 0.130 | 0.80(0.40-1.58) | 0.519 | 23/337 | 1.23(0.66-2.32) | 0.524 | 1.06(0.54-2.12) | 0.858 | 21/337 | 1.11(0.59-2.13) | 0.744 | 0.90(0.44-1.84) | 0.778 |
| CALLY, C-reactive protein-albumin-lymphocyte index; SII, Systemic Immune Inflammation Index; SIRI, System Inflammation Response Index; T, tertile; OR, Odds Ratio; HR, Hazard Ratio. Total Immune inflammation Index was calculated by the Immune inflammation Index at baseline plus the Immune inflammation Index at the second survey. | | | | | | | | | | | | | | | |

**Table S7. Association of total in Immune-Inflammation Index with stroke prognosis in 3-month**

|  | CALLY | | | | | SII | | | | | SIRI | | | | |
| --- | --- | --- | --- | --- | --- | --- | --- | --- | --- | --- | --- | --- | --- | --- | --- |
| Poor outcome | | | | | | | | | | | | | | | |
|  | Events, (%) | HR/OR  (95% CI) | P | HR/OR  (95% CI) | P | Events, (%) | HR/OR  (95% CI) | P | HR/OR  (95% CI) | P | Events, (%) | HR/OR  (95% CI) | P | HR/OR  (95% CI) | P |
| TotalT1 | 188/337 | 1(reference) |  | 1(reference) |  | 53/337 | 1(reference) |  | 1(reference) |  | 53/337 | 1(reference) |  | 1(reference) |  |
| TotalT2 | 79/337 | 0.24(0.17-0.34) | <0.001 | 0.48(0.31-0.72) | <0.001 | 91/337 | 1.98 (1.36-2.91) | <0.001 | 1.89(1.21-2.98) | 0.006 | 96/337 | 2.13(1.47-3.13) | <0.001 | 1.98(1.26-3.14) | 0.003 |
| TotalT3 | 50/337 | 0.14(0.09-0.20) | <0.001 | 0.31(0.20-0.48) | <0.001 | 173/337 | 5.65(3.96-8.18) | <0.001 | 2.75(1.02-2.32) | <0.001 | 168/337 | 5.33(3.73-7.71) | <0.001 | 2.85(1.80-4.53) | <0.001 |
| Death | | | | | | | | | | | | | | | |
| TotalT1 | 50/337 | 1(reference) |  | 1(reference) |  | 6/337 | 1(reference) |  | 1(reference) |  | 6/337 | 1(reference) |  | 1(reference) |  |
| TotalT2 | 3/337 | 0.05(0.01-0.14) | <0.001 | 0.09(0.02-0.28) | <0.001 | 9/337 | 1.51(0.54-4.56) | 0.436 | 1.73(0.58-5.56) | 0.331 | 8/337 | 1.34(0.46-4.11) | 0.59 | 1.32(0.43-3.41) | 0.634 |
| TotalT3 | 1/337 | 0.02(0.01-0.08) | <0.001 | 0.04(0.002-0.22) | 0.003 | 39/337 | 7.22(3.24-19.20) | <0.001 | 3.41(1.38-9.83) | 0.013 | 40/337 | 7.43(3.34-19.74) | <0.001 | 3.43(1.37-9.96) | 0.013 |
| Composite Vascular events | | | | | | | | | | | | | | | |
| TotalT1 | 16/337 | 1(reference) |  | 1(reference) |  | 5/337 |  |  |  |  | 7/337 | 1(reference) |  | 1(reference) |  |
| TotalT2 | 2/337 | 0.12(0.02-0.43) | 0.005 | 0.13(0.02-0.48) | 0.008 | 9/337 | 1.82(0.62-5.98) | 0.287 | 1.67(0.56-5.54) | 0.371 | 8/337 | 1.15(0.41-3.30) | 0.794 | 0.50(0.34-2.99) | 0.997 |
| TotalT3 | 7/337 | 0.435(0.16-1.01) | 0.063 | 0.51(0.18-1.34) | 0.182 | 11/337 | 2.24(0.81-7.18) | 0.139 | 1.64(0.54-5.6) | 0.399 | 10/337 | 1.44(0.55-4.01) | 0.463 | 1.34(0.36-3.18) | 0.934 |
| CALLY, C-reactive protein-albumin-lymphocyte index; SII, Systemic Immune Inflammation Index; SIRI, System Inflammation Response Index; T, tertile; OR, Odds Ratio; HR, Hazard Ratio. Total Immune inflammation Index was calculated by the Immune inflammation Index at baseline plus the Immune inflammation Index at the second survey. | | | | | | | | | | | | | | | |

**Table S8. Association of change in Immune-Inflammation Index with stroke prognosis in 1-year**

|  | CALLY | | | | | SII | | | | | SIRI | | | | |
| --- | --- | --- | --- | --- | --- | --- | --- | --- | --- | --- | --- | --- | --- | --- | --- |
| Poor outcome | | | | | | | | | | | | | | | |
|  | Events, (%) | HR/OR  (95% CI) | P | HR/OR  (95% CI) | P | Events, (%) | HR/OR  (95% CI) | P | HR/OR  (95% CI) | P | Events, (%) | HR/OR  (95% CI) | P | HR/OR  (95% CI) | P |
| △T1 | 118/337 | 1(reference) |  | 1(reference) |  | 84/337 | 1(reference) |  | 1(reference) |  | 94/337 | 1(reference) |  | 1(reference) |  |
| △T2 | 107/337 | 0.89(0.64-1.24) | 0.498 | 0.84(0.55-1.30) | 0.435 | 63/337 | 0.69(0.48-1.00) | 0.051 | 0.97(0.61-1.54) | 0.908 | 51/337 | 0.46(0.31-0.67) | <0.001 | 0.63(0.39-1.01) | 0.055 |
| △T3 | 92/337 | 0.68(0.48-0.96) | 0.03 | 0.62(0.40-0.97) | 0.037 | 126/337 | 1.80(1.29-2.51) | <0.001 | 1.61(1.06-2.47) | 0.027 | 128/337 | 1.58(1.15-2.19) | 0.005 | 1.48(0.97-2.26) | 0.068 |
| Death | | | | | | | | | | | | | | | |
| △T1 | 48/337 | 1(reference) |  | 1(reference) |  | 32/337 | 1(reference) |  | 1(reference) |  | 28/337 | 1(reference) |  | 1(reference) |  |
| △T2 | 28/337 | 0.56(0.34-0.91) | 0.021 | 0.53(0.31-0.92) | 0.025 | 19/337 | 0.57(0.31-1.02) | 0.061 | 0.71(0.37-1.35) | 0.308 | 11/337 | 0.37(0.17-0.74) | 0.007 | 0.52(0.24-1.10) | 0.095 |
| △T3 | 18/337 | 0.37(0.21-0.63) | <0.001 | 0.35(0.18-0.63) | <0.001 | 43/337 | 1.39(0.86-2.28) | 0.179 | 1.19(0.70-2.05) | 0.518 | 55/337 | 2.15(1.34-3.53) | 0.002 | 2.08(1.23-3.59) | 0.007 |
| Composite Vascular events | | | | | | | | | | | | | | | |
| △T1 | 18/337 | 1(reference) |  | 1(reference) |  | 22/337 |  |  |  |  | 22/337 | 1(reference) |  | 1(reference) |  |
| △T2 | 21/337 | 1.18(0.62-2.27) | 0.621 | 1.14(0.59-2.22) | 0.697 | 21/337 | 0.90(0.48-1.69) | 0.750 | 0.95(0.50-1.80) | 0.878 | 17/337 | 0.76(0.39-1.45) | 0.411 | 0.77(0.39-1.50) | 0.441 |
| △T3 | 24/337 | 1.36(0.73-2.59) | 0.341 | 1.32(0.70-2.55) | 0.391 | 20/337 | 0.95(0.51-1.77) | 0.875 | 0.89(0.47-1.68) | 0.719 | 24/337 | 1.10(0.60-2.01) | 0.760 | 1.04(0.56-1.93) | 0.909 |
| CALLY, C-reactive protein-albumin-lymphocyte index; SII, Systemic Immune Inflammation Index; SIRI, System Inflammation Response Index; T, tertile; OR, Odds Ratio; HR, Hazard Ratio. △T was calculated by the Immune inflammation Index at the second survey minus the Immune inflammation Index at baseline. | | | | | | | | | | | | | | | |

**Table S9. Association of change in Immune-Inflammation Index with stroke prognosis in 3-month**

|  | CALLY | | | | | SII | | | | | SIRI | | | | |
| --- | --- | --- | --- | --- | --- | --- | --- | --- | --- | --- | --- | --- | --- | --- | --- |
| Poor outcome | | | | | | | | | | | | | | | |
|  | Events, (%) | HR/OR  (95% CI) | P | HR/OR  (95% CI) | P | Events, (%) | HR/OR  (95% CI) | P | HR/OR  (95% CI) | P | Events, (%) | HR/OR  (95% CI) | P | HR/OR  (95% CI) | P |
| △T1 | 103/337 | 1(reference) |  | 1(reference) |  | 99/337 | 1(reference) |  | 1(reference) |  | 114/337 | 1(reference) |  | 1(reference) |  |
| △T2 | 94/337 | 0.87(0.63-1.21) | 0.414 | 0.81(0.53-1.23) | 0.313 | 76/337 | 0.70(0.49-0.99) | 0.044 | 1.00(0.65-1.54) | 0.983 | 65/337 | 0.47(0.33-0.66) | <0.001 | 0.62(0.40-0.96) | 0.034 |
| △T3 | 76/337 | 0.72(0.52-0.99) | 0.046 | 0.67(0.44-1.01) | 0.058 | 142/337 | 1.75(1.27-2.41) | <0.001 | 1.54(1.02-2.32) | 0.039 | 138/337 | 1.36(0.99-1.86) | 0.056 | 1.15(0.77-1.73) | 0.498 |
| Death | | | | | | | | | | | | | | | |
| △T1 | 33/337 | 1(reference) |  | 1(reference) |  | 16/337 | 1(reference) |  | 1(reference) |  | 19/337 | 1(reference) |  | 1(reference) |  |
| △T2 | 15/337 | 0.44(0.23-0.82) | 0.011 | 0.43(0.21-0.86) | 0.019 | 10/337 | 0.61(0.27-1.35) | 0.234 | 0.82(0.32-1.99) | 0.659 | 6/337 | 0.30(0.11-0.73) | 0.012 | 0.43(0.14-1.12) | 0.100 |
| △T3 | 6/337 | 0.20(0.08-0.44) | <0.001 | 0.14(0.05-0.35) | <0.001 | 28/337 | 1.82(0.98-3.50) | 0.065 | 1.63(0.81-3.40) | 0.177 | 29/337 | 1.58(0.87-2.91) | 0.137 | 1.38(0.71-2.74) | 0.348 |
| Composite Vascular events | | | | | | | | | | | | | | | |
| △T1 | 7/337 | 1(reference) |  | 1(reference) |  | 7/337 | 1(reference) |  | 1(reference) |  | 9/337 | 1(reference) |  | 1(reference) |  |
| △T2 | 10/337 | 1.44(0.55-4.01) | 0.463 | 1.48(0.55-4.18) | 0.439 | 8/337 | 1.15(0.41-3.30) | 0.794 | 1.37(0.48-4.03) | 0.556 | 4/337 | 0.44(0.12-1.36) | 0.173 | 0.50(0.13-1.59) | 0.260 |
| △T3 | 8/337 | 1.15(0.41-3.30) | 0.794 | 1.17(0.41-3.41) | 0.770 | 10/337 | 1.44(0.55-4.01) | 0.463 | 1.37(0.51-3.87) | 0.537 | 12/337 | 1.35(0.56-3.34) | 0.507 | 1.34(0.55-3.36) | 0.526 |
| CALLY, C-reactive protein-albumin-lymphocyte index; SII, Systemic Immune Inflammation Index; SIRI, System Inflammation Response Index; T, tertile; OR, Odds Ratio; HR, Hazard Ratio. △T was calculated by the Immune inflammation Index at the second survey minus the Immune inflammation Index at baseline. | | | | | | | | | | | | | | | |

**Table S10. Association of total in Immune-Inflammation Index with stroke prognosis in 1-year**

|  | CALLY | | | | | SII | | | | | SIRI | | | | |
| --- | --- | --- | --- | --- | --- | --- | --- | --- | --- | --- | --- | --- | --- | --- | --- |
| Poor outcome | | | | | | | | | | | | | | | |
|  | Events, (%) | HR/OR  (95% CI) | P | HR/OR  (95% CI) | P | Events, (%) | HR/OR  (95% CI) | P | HR/OR  (95% CI) | P | Events, (%) | HR/OR  (95% CI) | P | HR/OR  (95% CI) | P |
| Decrease group | 55/80 | 1(reference) |  | 1(reference) |  | 56/270 | 1(reference) |  | 1(reference) |  | 62/309 | 1(reference) |  | 1(reference) |  |
| Stable group | 48/200 | 0.14(0.08-0.25) | <0.001 | 0.20(0.09-0.42) | <0.001 | 93/392 | 1.19(0.82-1.74) | 0.367 | 1.54(0.96-2.48) | 0.077 | 36/125 | 1.61(0.99-2.59) | 0.050 | 1.95(0.90-3.07) | 0.103 |
| Increase group | 170/731 | 0.14(0.08-0.23) | <0.001 | 0.15(0.08-0.30) | <0.001 | 124/349 | 2.11(1.29-2.51) | <0.001 | 1.94(1.23-3.11) | 0.005 | 175/577 | 1.73(1.25-2.43) | 0.001 | 2.69(1.18-2.75) | 0.007 |
| Death | | | | | | | | | | | | | | | |
| Decrease group | 34/80 | 1(reference) |  | 1(reference) |  | 15/270 | 1(reference) |  | 1(reference) |  | 9/309 | 1(reference) |  | 1(reference) |  |
| Stable group | 11/200 | 0.08(0.04-0.16) | <0.001 | 0.11(0.05-0.25) | <0.001 | 35/392 | 1.67(0.91-3.20) | 0.11 | 1.80(0.93-3.66) | 0.09 | 19/125 | 4.20(1.79-10.35) | 0.001 | 4.53(1.81-11.87) | 0.001 |
| Increase group | 49/731 | 0.10(0.06-0.17) | <0.001 | 0.12(0.06-0.22) | <0.001 | 44/349 | 2.45(1.36-4.65) | 0.004 | 2.15(1.14-4.27) | 0.022 | 71/577 | 4.68(2.43-10.17) | <0.001 | 4.71(2.35-10.59) | <0.001 |
| Composite Vascular events | | | | | | | | | | | | | | | |
| Decrease group | 6/80 | 1(reference) |  | 1(reference) |  | 15/270 | 1(reference) |  | 1(reference) |  | 19/309 | 1(reference) |  | 1(reference) |  |
| Stable group | 11/200 | 0.72(0.26-2.15) | 0.528 | 0.92(0.32-2.87) | 0.878 | 21/392 | 1.26(0.66-2.47) | 0.49 | 1.32(0.69-2.63) | 0.406 | 6/125 | 0.77(0.27-1.87) | 0.586 | 0.65(0.23-1.60) | 0.373 |
| Increase group | 46/731 | 0.83(0.37-2.22) | 0.676 | 0.96(0.41-2.67) | 0.933 | 21/349 | 1.09(0.55-2.19) | 0.808 | 1.02(0.51-2.07) | 0.959 | 38/577 | 1.08(0.62-1.94) | 0.801 | 0.97(0.55-1.77) | 0.927 |
| CALLY, C-reactive protein-albumin-lymphocyte index; SII, Systemic Immune Inflammation Index; SIRI, System Inflammation Response Index; T, tertile; OR, Odds Ratio; HR, Hazard Ratio. The increase group is defined as having an increase of more than 10% in the second measurement compared to the first. The stable group is defined as having a fluctuation between -10% and 10% in the second measurement compared to the first. The decrease group is defined as having a decrease of more than 10% in the second measurement compared to the first. | | | | | | | | | | | | | | | |

**Table S11. Association of change in Immune-Inflammation Index with stroke prognosis in 3-month**

|  | CALLY | | | | | SII | | | | | SIRI | | | | |
| --- | --- | --- | --- | --- | --- | --- | --- | --- | --- | --- | --- | --- | --- | --- | --- |
| Poor outcome | | | | | | | | | | | | | | | |
|  | Events, (%) | HR/OR  (95% CI) | P | HR/OR  (95% CI) | P | Events, (%) | HR/OR  (95% CI) | P | HR/OR  (95% CI) | P | Events, (%) | HR/OR  (95% CI) | P | HR/OR  (95% CI) | P |
| Decrease group | 55/80 | 1(reference) |  | 1(reference) |  | 72/270 | 1(reference) |  | 1(reference) |  | 83/309 | 1(reference) |  | 1(reference) |  |
| Stable group | 63/200 | 0.21(0.12-0.36) | <0.001 | 0.34(0.16-0.71) | 0.004 | 104/392 | 0.70 (0.49-0.99) | 0.969 | 1.19(0.77-1.86) | 0.439 | 44/125 | 1.48(0.94-2.30) | 0.085 | 1.98(1.26-3.14) | 0.117 |
| Increase group | 199/731 | 0.17(0.10-0.28) | <0.001 | 0.20(0.10-0.39) | <0.001 | 141/349 | 1.53(1.09-2.14) | <0.001 | 1.66(1.08-2.58) | 0.023 | 190/577 | 1.34(0.99-1.82) | 0.063 | 2.85(1.80-4.53) | 0.359 |
| Death | | | | | | | | | | | | | | | |
| Decrease group | 26/80 | 1(reference) |  | 1(reference) |  | 8/270 | 1(reference) |  | 1(reference) |  | 4/309 | 1(reference) |  | 1(reference) |  |
| Stable group | 6/200 | 0.06(0.02-0.15) | <0.001 | 0.12(0.04-0.34) | <0.001 | 17/392 | 1.48(0.65-3.69) | 0.365 | 1.69(0.67-4.62) | 0.281 | 10/125 | 6.63(2.17-24.56) | 0.002 | 7.48(2.21-30.07) | 0.002 |
| Increase group | 22/731 | 0.06(0.03-0.12) | <0.001 | 0.09(0.04-0.19) | <0.001 | 29/349 | 2.97(1.40-7.06) | 0.008 | 2.71(1.17-6.99) | 0.027 | 40/577 | 5.68(2.26-19.05) | 0.001 | 5.56 (2.07-19.55) | 0.002 |
| Composite Vascular events | | | | | | | | | | | | | | | |
| Decrease group | 3/80 | 1(reference) |  | 1(reference) |  | 6/270 | 1(reference) |  | 1(reference) |  | 6/309 | 1(reference) |  | 1(reference) |  |
| Stable group | 3/200 | 0.39(0.07-2.15) | 0.256 | 0.50(0.09-2.92) | 0.419 | 9/392 | 1.03(0.37-3.12) | 0.95 | 1.14(0.40-3.48) | 0.812 | 8/125 | 1.24(0.26-4.79) | 0.762 | 1.19(0.24-4.67) | 0.812 |
| Increase group | 19/731 | 0.68(0.23-2.96) | 0.550 | 0.85(0.26-3.93) | 0.811 | 10/349 | 1.30(0.48-3.86) | 0.618 | 1.24(0.44-3.74) | 0.691 | 16/577 | 1.44(0.59-4.05) | 0.451 | 1.41(0.56-4.01) | 0.489 |
| CALLY, C-reactive protein-albumin-lymphocyte index; SII, Systemic Immune Inflammation Index; SIRI, System Inflammation Response Index; T, tertile; OR, Odds Ratio; HR, Hazard Ratio. The increase group is defined as having an increase of more than 10% in the second measurement compared to the first. The stable group is defined as having a fluctuation between -10% and 10% in the second measurement compared to the first. The decrease group is defined as having a decrease of more than 10% in the second measurement compared to the first. | | | | | | | | | | | | | | | |

**TableS12. Association of change in Immune-Inflammation Index with stroke prognosis in 1-year by sex**

|  |  | CALLY | | | SII | | | SIRI | | |
| --- | --- | --- | --- | --- | --- | --- | --- | --- | --- | --- |
|  |  | Events, (%) | HR/OR (95% CI) | P | Events, (%) | HR/OR  (95% CI) | P | Events, (%) | HR/OR  (95% CI) | P |
| Poor outcome | | | | | | | | | | |
| Male | △T1 | 63/222 | 1(reference) |  | 44/229 | 1(reference) |  | 44/216 | 1(reference) |  |
|  | △T2 | 56/222 | 0.66(0.38-1.14) | 0.139 | 36/212 | 1.36(0.75-2.48) | 0.304 | 35/209 | 1.21(0.66-2.23) | 0.533 |
|  | △T3 | 44/222 | 0.52(0.30-0.92) | 0.024 | 83/225 | 2.29(1.34-3.98) | 0.003 | 84/241 | 2.06(1.20-3.57) | 0.009 |
| Female | △T1 | 39/115 | 1(reference) |  | 22/108 | 1(reference) |  | 30/129 | 1(reference) |  |
|  | △T2 | 38/115 | 0.99(0.56-1.64) | 0.630 | 37/124 | 2.13(0.96-4.86) | 0.065 | 36/120 | 1.82(0.83-4.06) | 0.139 |
|  | △T3 | 33/115 | 0.90(0.57-0.99) | 0.045 | 51/113 | 2.77(1.27-6.21) | 0.012 | 44/96 | 2.35(1.08-5.24) | 0.033 |
| Death | | | | | | | | | | |
| Male | △T1 | 25/222 | 1(reference) |  | 9/229 | 1(reference) |  | 7/216 | 1(reference) |  |
|  | △T2 | 20/222 | 0.69(0.33-1.39) | 0.295 | 16/212 | 3.22(1.27-8.79) | 0.017 | 13/209 | 3.06(1.12-9.14) | 0.034 |
|  | △T3 | 9/222 | 0.28(0.11-0.69) | 0.005 | 29/225 | 3.21(1.39-8.09) | 0.009 | 34/241 | 5.13(2.16-13.89) | <0.001 |
| Female | △T1 | 22/115 | 1(reference) |  | 5/108 | 1(reference) |  | 9/129 | 1(reference) |  |
|  | △T2 | 8/115 | 0.27(0.09-0.75) | 0.016 | 13/124 | 2.81(0.84-10.74) | 0.106 | 10/120 | 1.67(0.54-5.39) | 0.374 |
|  | △T3 | 10/115 | 0.3(0.10 -0.81) | 0.022 | 22/113 | 5.02(1.67-17.97) | 0.007 | 21/96 | 3.24(1.19-9.62) | 0.026 |
| Composite Vascular events | | | | | | | | | | |
| Male | △T1 | 11/115 | 1(reference) |  | 10/217 | 1(reference) |  | 11/216 | 1(reference) |  |
|  | △T2 | 13/115 | 1.02(0.44-2.43) | 0.962 | 12/213 | 1.30(0.54-3.20) | 0.562 | 13/209 | 1.25(0.53-2.99) | 0.612 |
|  | △T3 | 16/115 | 1.31(0.58-3.02) | 0.523 | 18/236 | 1.69(0.76-3.96) | 0.208 | 16/241 | 1.27(0.57-2.93) | 0.567 |
| Female | △T1 | 7/115 | 1(reference) |  | 9/108 | 1(reference) |  | 11/129 | 1(reference) |  |
|  | △T2 | 8/115 | 1.29(0.43-4.00) | 0.651 | 10/124 | 0.99(0.36-2.74) | 0.991 | 4/120 | 0.25(0.06-0.83) | 0.033 |
|  | △T3 | 8/115 | 1.27(0.42-3.96) | 0.673 | 4/113 | 0.32(0.08-1.08) | 0.081 | 8/96 | 0.82(0.28-2.27) | 0.706 |
| CALLY, C-reactive protein-albumin-lymphocyte index; SII, Systemic Immune Inflammation Index; SIRI, System Inflammation Response Index; T, tertile; OR, Odds Ratio; HR, Hazard Ratio. △T was calculated by the Immune-Inflammation index at the second survey minus the Immune inflammation Index at baseline. | | | | | | | | | | |

**Table S13. Association of change in Immune-Inflammation Index with stroke prognosis in 1-year by age**

|  |  | CALLY | | | SII | | | SIRI | | |
| --- | --- | --- | --- | --- | --- | --- | --- | --- | --- | --- |
|  |  | Events, (%) | HR/OR (95% CI) | P | Events, (%) | HR/OR  (95% CI) | P | Events, (%) | HR/OR  (95% CI) | P |
| Poor outcome | | | | | | | | | | |
| ≥70 | △T1 | 64/145 | 1(reference) |  | 50/153 | 1(reference) |  | 45/148 | 1(reference) |  |
|  | △T2 | 59/153 | 0.81(0.44-1.48) | 0.491 | 45/133 | 1.07(0.47-1.64) | 0.683 | 44/138 | 1.01(0.66-1.23) | 0.326 |
|  | △T3 | 54/152 | 0.73(0.40-0.97) | 0.039 | 82/164 | 1.87(1.13-3.12) | 0.016 | 88/164 | 1.85(1.02-3.10) | 0.038 |
| <70 | △T1 | 54/192 | 1(reference) |  | 24/184 | 1(reference) |  | 27/189 | 1(reference) |  |
|  | △T2 | 47/184 | 0.90(0.47-1.71) | 0.737 | 28/204 | 1.00(0.49-2.04) | 0.991 | 29/199 | 1.02(0.38-1.51) | 0.139 |
|  | △T3 | 30/185 | 0.53(0.26-0.97) | 0.041 | 44/173 | 1.49(1.07-2.88) | 0.041 | 40/173 | 1.34(1.08-5.24) | 0.033 |
| Death | | | | | | | | | | |
| ≥70 | △T1 | 34/145 | 1(reference) |  | 22/153 | 1(reference) |  | 15/148 | 1(reference) |  |
|  | △T2 | 23/153 | 0.61(0.31-1.18) | 0.143 | 21/133 | 1.02(0.47-1.51) | 0.426 | 17/138 | 1.06(0.62-2.14) | 0.215 |
|  | △T3 | 18/152 | 0.48(0.23-0.96) | 0.042 | 32/164 | 1.20(0.66-2.18) | 0.550 | 43/164 | 1.89(1.01-3.63) | 0.045 |
| <70 | △T1 | 13/192 | 1(reference) |  | 3/184 | 1(reference) |  | 3/189 | 1(reference) |  |
|  | △T2 | 5/184 | 0.29(0.08-0.89) | 0.040 | 5/204 | 1.54(0.60-3.74) | 0.153 | 4/199 | 1.23(0.79-2.45) | 0.315 |
|  | △T3 | 1/185 | 0.05(0.01-0.16) | 0.004 | 11/173 | 1.95(0.63-6.78) | 0.263 | 12/173 | 3.58(1.11-13.71) | 0.042 |
| Composite Vascular events | | | | | | | | | | |
| ≥70 | △T1 | 9/145 | 1(reference) |  | 9/153 | 1(reference) |  | 11/148 | 1(reference) |  |
|  | △T2 | 10/153 | 1.00(0.38-2.63) | 0.997 | 14/133 | 2.06(0.85-5.25) | 0.114 | 8/138 | 0.76(0.28-2.00) | 0.579 |
|  | △T3 | 14/152 | 1.54(0.64-3.91) | 0.345 | 10/164 | 1.00(0.38-2.65) | 0.996 | 14/164 | 1.16(0.50-2.75) | 0.736 |
| <70 | △T1 | 9/192 | 1(reference) |  | 13/184 | 1(reference) |  | 11/189 | 1(reference) |  |
|  | △T2 | 11/184 | 1.13(0.44-2.94) | 0.805 | 6/204 | 0.42(0.14-1.12) | 0.057 | 9/199 | 0.74(0.28-1.90) | 0.527 |
|  | △T3 | 10/185 | 0.98(0.37-2.59) | 0.959 | 11/173 | 0.84(0.34-1.99) | 0.121 | 10/173 | 0.95(0.37-2.38) | 0.906 |
| CALLY, C-reactive protein-albumin-lymphocyte index; SII, Systemic Immune Inflammation Index; SIRI, System Inflammation Response Index; T, tertile; OR, Odds Ratio; HR, Hazard Ratio. △T was calculated by the Immune inflammation Index at the second survey minus the Immune inflammation Index at baseline. | | | | | | | | | | |
